# Supplementary material for: Learning curve and surgical outcome of robotic assisted colorectal surgery with ERAS program
Source: Sci Rep. 2022 Nov 29;12:20566. doi: 10.1038/s41598-022-24665-w (PMC9709162; doi:10.1038/s41598-022-24665-w)
Supplement: Supplementary file 1 — Supplementary Information 1. [file 41598_2022_24665_MOESM1_ESM.zip › LSC Raw data-Ñ[▒K/115/H_ñw╝╨░O▒Kñσ.pdf]

正式摘要(電子簽章：00000211228268)	□【讀取】憑證	
W52 / W52　□英文標題(注意:無英文姓名-請補病人基本檔!)		

臺中榮民總醫院
出院病歷摘要

轉入號 : 轉出號 :

[redacted]

(8)流水編號	
(9)入院日期	110/01/20 (2021) 直腸外科 W52 - 162
(10)轉科日期	
(11)出院日期	110/02/27 (2021) 住院天數計 38 日

診斷

- 入院 : 1.transverse colon adenocarcinoma
  2.hypertension
  3.old CVA
  4.BPH
- 出院 : 1. T-colon adenocarcinoma, pT4aN1aM0, Stage IIIB, s/p Right hemicolectomy with anastomosis on 2021/01/28
  2. Hypertension
  3. CVA
  4. BPH
  5. Moderate-severe AR with aortic root dilation

主訴

- poor appetite, body weight loss since July 2020

病史

- This 88 years old male patient has past history of hypertension, CVA, BPH, cataract. He lived in nursing home of 二林基督教醫院. On July 2020, poor appetite, general weakness and body weight loss were found. Tarry stool was also noted. Lab data showed normocytic anemia. UGI and LGI scope were arranged. Ulcerative tumor at transverse colon, 100cm from anal verge was found and adenocarcinoma was proved in biopsy. He was brought to our hospital for second opinion on Sep. 2020. PET that showed no distal metastasis. Operation was not suggested due to poor nutrition status so he took Xeloda initially. General status improved in recent months. Thus, he was admitted for surgical treatment.

體檢發現

-
  - General appearance :
    - easy looking , conscious clear , without cardiopulmonary distress
    - Vital signs自動帶入date 體溫(BT) 脈搏(P) 呼吸(R) 血壓(BP) 身高(BH) 體重(BW) BMI
      20210120 37.1℃ 112次/分 16次/分 157/74mmHg
  - HEENT :
    - Eyes: no anemic conjunctiva, no icteric sclera, normal EOM
    - ENT: no otorrhea, no rhinorrhea, no erythematous mucosa, no injected tonsil, no deformity
  - Neck: supple, freely movable, trachea midline,no deformity, no engorged jugular vein , no palpable thyroid or lymph node enlargement
  - Chest:
    - Inspection: Grossly normal, symmetric and fully expansion, no accessory muscle used, no operation scar, no spider angiomata, no gynecomastia
    - Palpation: no palpable mass or nodule, no subcutaneous emphysema, no tenderness
    - Ausculation: clear breathing sound
  - Heart:
    - Percussion: normal heart size
    - Ausculation: regular heart beat, normal S1, S2, no gallop, no murmur
  - Abdomen:
    - Inspection: in

-
  - ▪ flat contour, symetrically, no scar, no stria, no dilated veins, no rashes
  - Ausculation: normoactive bowel sound, no abdominal bruits
  - palpation: soft, no muscle guarding or rigidity, no local tenderness, no rebounding pain, no palpable mass, no palpable pulsation
  - Percussion: no shifting dulllness, no hepatomegaly, no splenomagaly
-

Peripheral pulsation :	CA	BA	RA	FA	PA	PTA	DPA
Right side :	++	++	++	++	++	++	++/+++
Left side :	++	++	++	++	++	++	++/+++

- Genito-anus: no tenderness or palpable mass lesion or rectal shelf, normal external genitalia, normal anal tone, brown stool

CRS CONDITION

- Colonoscope report: ulcertive tumor, 100cm FAV
- CEA: 9.5 ng/ml
- Imaging studies(Sono , CT of abdomen and chest Films):
  - 1. Collapse status of colon. Subtle lesion may not be well identified. Suggest correlation with clinical profile and further evaluation if clinically indicated.2. No enlarged (short-axis diameter ＞1 cm) lymph nodes.3. Nodules in right lower lung. Nature to be determined.Other incidental findings were described in above text.TX Primary tumor cannot be assessedN0 No regional lymph node metastasisMX
-
  - Lung

手術日期及方法

- 手術日期及方法20210128 Laparoscopic right colectomy and anastomosis
  Side to side anastomsis with GIA 80/3.8mmx2
  Surgiwrap +

住院治療經過

- This 88 years old male patient has past history of hypertension, CVA, BPH, cataract. He lived in nursing home of 二林基督教醫院. On July 2020, poor appetite, general weakness and body weight loss were found. Tarry stool was also noted. Lab data showed normocytic anemia. UGI and LGI scope were arranged. Ulcerative tumor at transverse colon, 100cm from anal verge was found and adenocarcinoma was proved in biopsy. He was brought to our hospital for second opinion on Sep. 2020. PET that showed no distal metastasis. Operation was not suggested due to poor nutrition status so he took Xeloda initially. General status improved in recent months. Thus, he was admitted for surgical treatment.
  After admission, pre-OP survey including 2D echo, lung function test. Moderate-severe AR with aortic root dilatation was found. Lung function test was not complete due to poor compliance. Parenteral nutrition was given. We also consulted SICU for possible difficult weaning ventilator. Operation with laparoscopic right hemicolectomy was done on 1/28 and the patient was transferred to SICU for post-OP care.
  penrose was removed on 2/3, diet was tried on 2/2. However due to poor digestion condition, erythromycin, primparan was add. Anal tube was used on 2/5. Extubation was done on 2/09 and the patient tolerate well under N/C.he diet was gradually increased from trying water to now 1000kcal today. The general condition was smooth, he was transferred to ordinary ward. Under stable condition, the patient will be discharged

合併症

一般檢查

- 累積報告 :

DATE	TIME	ALB	TP	BIL.T	BIL.D	ALKP	AST	ALT	LDH	CHOL	TG	HDL-C	LDL	UA	NH3	AMY
1100120	1530	3.5		0.36	0.11	78	19	7	178							
1100126	0849									186	102					
1100127	1050	3.2		0.23	0.08		16	5								
1100128	2047			0.31	0.10											
1100128	2114						16	9								
1100130	0510	2.6														
1100131	0509			0.30	0.11	54	40	7								
1100204	0502			0.31	0.15	37	18	3								
1100207	0557	2.9		0.38	0.17		23	3	211							
1100209	0441			0.36	0.17	48	21	4								
1100212	0548	3.0		0.43	0.19	118	26	6								
1100217	0511			0.35	0.16	177	36	8								

1100224	0901			0.41	0.18		35	12								
1100225	0841	3.5														

DATE	TIME	WBC	RBC	HGB	HCT	MCV	PLT	BLST	NEUT	BAND	SEG	LYM	MON	EOS	BAS
1100120	1514	5010	3.90	11.3	36.1	92.6	234		70.8			20.0	7.2	1.8	0.2
1100127	1051	5330	3.90	11.5	36.3	93.1	201		73.7			16.9	6.6	2.4	0.4
1100128	2102	7820	3.27	9.5	30.3	92.7	192		83.5			12.7	3.3	0.4	0.1
1100131	0507	5610	2.55	7.2	23.8	93.3	157		77.5			13.2	6.6	2.5	0.2
1100201	0519	6550	3.33	9.3	29.3	88.0	199		83.3			7.2	6.9	2.4	0.2
1100203	0515	5890		9.3	29.5		203		76.2			10.0	9.8	3.7	0.3
1100204	0500	7170	3.25	9.4	29.3	90.2	212				87.0	6.0	6.0	1.0	0.0
1100207	0616	7570		10.0	31.0		271				74.0	12.0	12.0	2.0	0.0
1100209	0440	10770		9.6	28.7		266		84.0			9.7	4.2	1.9	0.2
1100212	0547	7840		9.8	29.9		387		79.0			10.6	7.3	2.8	0.3
1100217	0510	7210		9.1	28.5		383		73.1			13.9	8.6	3.7	0.7
1100224	0909	7470		9.2	26.9	84.6	282				71.0	16.0	6.0	4.0	1.0

DATE	TIME	NA	K	CL	CA	BUN	CREAT	Mg	P	CRP	ESR	PCT	LIPASE	LACTATE
1100120	1530	140	4.1	101	8.3	21	0.84							
1100127	1050	138	4.0	99	8.3	17	0.80							
1100128	2047	137	3.6				0.63							
1100128	2113													15.7
1100128	2114				7.9	13								
1100131	0509	133	3.3		7.0	18	0.75							
1100201	0533	135	3.6		7.0									
1100203	0514													7.4
1100203	0516	136	3.3	99	7.4	13	0.58							
1100203	1133							1.9	1.7					
1100204	0502	135	3.8		7.6	14	0.56							
1100205	0530								2.1					
1100207	0345													6.1
1100207	0557	133	4.6	100	7.5	14	0.61	2.1	3.0					
1100209	0441	127	4.8	97	7.6	16	0.59							
1100209	0443													6.3
1100209	1706	129					0.55							
1100210	1136	136	4.2											
1100212	0548	132	4.3	98	7.6	20	0.58	1.9	2.9					
1100214	0450	137	4.5											
1100217	0511	133	4.8	101	7.9	18	0.65							
1100224	0901	126	5.4			19	0.57							
1100225	0841	128	5.3	94	7.9									

DATE	TIME	GLUCOSE	PAB	TRF	ZN	CU	KETONE	CK	CKMB	PT-P	PT-C	APTT-P	APTT-C	TRO-T
1100120	1514									10.9	10.3	29.5		
1100120	1529	104												

DATE	TIME	SP-GR	PH	PROT	GLUC	KETON	BILI	UROB	OB	NITR	LEU	RBC	WBC	EPCEL
1100128	2114	1.018	7.5	1+	250.0	Negative	Negative	0.2	1+	Positive	Trace	10-20	2-5	0-1
1100201	1942	1.017	7.0	2+	Negative	Negative	Negative	0.2	2+	Positive	1+	10-20	2-5	0-1
1100208	1426	1.016	7.5	2+	Negative	Negative	Negative	0.2	3+	Negative	Negative	3+	5-10	0-1

DATE	TIME	STOOL-OB	FAT	RBC	WBC	PARA
1100219	0819			0 - 2	0 - 2	Not Found

DATE	TIME	PH	PCO2	PO2	SO2	BEB	HCO3	TCO2	HGB	FIO2
1100128	2046	7.335	46.4	443.4	100.0	-2.0	24.2	25.6		
1100131	0508	7.381	48.2	180.5	99.6	2.1	28.0	29.4		
1100204	0459	7.455	45.2	165.4	99.6	6.2	31.0	32.4		
1100211	0509	7.460	37.7	98.8	98.1	2.5	26.2	27.3		

特殊檢查

病理報告

- 活體組織切片病理報告　手術日期：**110/01/28**　病理號：**S1003631**

診斷醫師:李欣倪 報告日期:110/02/04
Pathologic diagnosis:
Colon, A-T, LAPA-RH--- Adenocarcinoma, moderately differentiated, arising in a tubulovillous adenoma, with focal extracellular mucin production, of transverse colon. Tumor invaded to serosa and metastasized to mesocolic lymph nodes (1/19). The appendix, ileocecal valve and bilateral cut ends are free of tumor.

- Ancillary study for diagnosis:
  IHC stains for MLH1, MSH2, PMS2 and MSH6 are done on section H. All above markers are preserved.
  Prognostic and predictive factor:
  1. Tumor size: 5 x 4 cm.
  2. Depth of invasion: Visceral peritoneum (including tumor continuous with serosal surface through area of inflammation).
  3. Histologic grade: G2: Moderately differentiated.
  4. Angiolymphatic invasion: Not identified.
  5. Perineural invasion: Not identified.
  6. Tumor deposits (discontinuous extramural extension): Present, specify number of deposits: 2.
  7. Macroscopic tumor perforation: Not identified.
  8. Surgical Margins: a) Proximal Margin: Uninvolved by invasive carcinoma, distance of tumor from margin: 17 cm. b) Distal Margin: Uninvolved by invasive carcinoma, distance of tumor from margin: 3 cm. c) Radial or Mesenteric Margin: Uninvolved by invasive carcinoma, distance of tumor from margin: 1 cm.
  9. Lymph node status: Involved (1/19).
  10. Extranodal involvement: Not identified.
  11. Tumor regression grading S/P CCRT: No known presurgical therapy.
  12. Type of polyp in which invasive carcinoma arose: Tubulovillous adenoma.
  13. Additional pathologic findings: Not identified.
  14. Pathological TNM Stage: pT4aN1a(According to the eighth edition, American Joint Committee on Cancer Staging Guidelines for Tumors).
  15. TNM descriptors: Not applicable.

其它

- 隔離註記:接觸防護【VRE(檢)(20210219)】

出院時情況

- 3.改門診治療

出院指示

出院計劃

主治醫師_2062F 蔣鋒帆_ / 住院醫師_2332G 林志安_

正式摘要**(電子簽章：000000211228268)**

	W52 / W52	

doctorRank:B
